# Dysregulated circulating miR-4429 serves as a novel non-invasive biomarker and is correlated with EGFR mutation in patients with non-small cell lung cancer

**DOI:** 10.17305/bjbms.2021.6450

**Published:** 2022-02-15

**Authors:** Mei Ruan, Liyan Sun, Wen Qiu, Yanjie Dong, Chunmei Fang, Haiyan Cui, Jiansheng Rong

**Affiliations:** 1 Department of Oncology, The Fourth People Hospital of Zibo, Zibo, Shandong, China; 2 Department of Gynecology, Zibo Lianchi Women and Infants Hospital, Zibo, Shandong, China; 3 Department of Pathology, The Fourth People Hospital of Zibo, Zibo, Shandong, China; 4 Department of Pathology, Zibo Central Hospital, Zibo, Shandong, China

**Keywords:** miR-4429, *EGFR*, non-small cell lung cancer, diagnosis, prognosis, *EGFR* mutation

## Abstract

This study aimed to investigate the correlation between microRNA (miR)-4429 and epidermal growth factor receptor (*EGFR*), the expression, and clinical significance of miR-4429 in patients with non-small cell lung cancer (NSCLC), and the relationship between miR-4429 and *EGFR* mutation in NSCLC patients. Blood samples were collected from 122 NSCLC patients and 72 healthy volunteers. miR-4429 expression and *EGFR* mRNA expression were detected by real-time quantitative polymerase chain reaction. Correlation between miR-4429 and *EGFR* was evaluated by dual-luciferase reporter assay and the Pearson correlation analysis. The ability of serum miR4429 to discriminate between NSCLC patients and healthy controls, and to discriminate between *EGFR* wild-type (*EGFR*-W) and *EGFR* mutant-type (*EGFR*-M) patients was assessed using receiver operating characteristic analysis. The relationship between miR-4429 and NSCLC patients’ survival was identified by KaplanMeier survival curves and log-rank test. The prognostic value of miR-4429 in NSCLC patients was evaluated by Cox regression analysis. miR-4429 could directly bind to *EGFR*. Serum miR-4429, decreased in NSCLC patients, was negatively correlated with serum *EGFR* mRNA expression in NSCLC patients. In addition, miR-4429 had a high diagnostic value for screening NSCLC patients from healthy controls, and was independently correlated with survival prognosis of NSCLC patients. Moreover, miR4429 was decreased in *EGFR*-M patients, which had a certain screening ability for *EGFR*M patients. Our findings indicate that miR-4429 is negatively correlated with *EGFR* in NSCLC, and may function as a diagnostic and prognostic biomarker for NSCLC patients. Additionally, miR-4429 is associated with *EGFR* mutation in NSCLC patients.

## INTRODUCTION

Lung cancer is a malignant tumor with high morbidity and mortality [1]. Non-small cell lung cancer (NSCLC) is the major subtype of lung cancer, accounting for 85% of lung cancer cases [2]. Despite current advances in diagnostic and therapeutic approaches, the prognosis of lung cancer remains poor with very low survival rates [3-5]. Therefore, early diagnosis and accurate prognosis are urgent issues in the treatment of NSCLC. Epidermal growth factor receptor (*EGFR*) is the major and well-studied oncogene of NSCLC [6]. Targeting *EGFR* is an important method of NSCLC treatment. The *EGFR* mutation is an important predictor of the effectiveness of targeted drug therapy with tyrosine kinase inhibitors (TKIs). However, drug resistance still occurs in more than 50% of patients [7]. Therefore, probing the molecules related to *EGFR* is expected to unearth more key molecules in the pathological mechanism of NSCLC, providing new ideas and new targets for the treatment of this disease.

MicroRNAs (miRs) are a type of non-protein coding RNAs that negatively regulate gene expression mainly by inducing targeted messenger RNA (mRNA) degradation or inhibiting targeted mRNA translation [8,9]. miRs have been found to play key roles in the pathogenesis of various diseases [10-12]. Many miRs such as miR-340 [13] and miR-142-3p [14] have been found to be involved in the pathogenesis of NSCLC. In addition, miR-4429 has been found to be downregulated and to inhibit tumor progression in some human malignancies such as ovarian cancer [15], cervical cancer [16], and gastric cancer [17]. In this study, we performed bioinformatics analysis prediction, and the results suggested that the 3’-untranslated region (3’-UTR) of *EGFR* had the binding sequence of miR-4429. However, the expression profile of miR-4429 in NSCLC, the relationship of miR-4429 with *EGFR* expression, and *EGFR* mutation in NSCLC patients are unknown.

Thus, the aim of this study was to explore whether miR-4429 was related to *EGFR*, measure the expression of miR-4429 in NSCLC, investigate its clinical value in NSCLC, and explore its relationship with *EGFR* mutation in NSCLC patients. This study is expected to provide a novel biomarker for the diagnosis and survival prognosis of NSCLC patients, and a novel target for the therapy in NSCLC patients.

## MATERIALS AND METHODS

### Study population and serum collection

Venous blood samples were collected from 122 NSCLC patients enrolled in The Fourth People Hospital of Zibo from 2014 to 2019. Blood samples of 72 healthy volunteers who underwent health physical examination during the same period were also collected as controls. Then, serum separation was performed by centrifugation immediately after blood sample collection to avoid hemolysis, and the serum was immediately stored at −80°C for further use. The inclusion criteria for NSCLC patients were: (1) they had not received any anti-tumor treatment before surgery; (2) they were diagnosed with NSCLC on pathological basis and (3) their clinical medical record data was fully available. Patients were excluded from this study if they: (1) presented with concomitant major organ diseases; (2) had renal, hepatic, or severe cardiac dysfunction; (3) complicated with other malignancies; (4) had incomplete clinical data. The information of clinicopathological characteristics of patients were recorded, including age (was dichotomized according to the age cut-off standard for elderly in China), gender, smoking, tumor size, *EGFR* mutation, lymph node metastasis, tumor node metastasis (TNM) stage, and histological subtypes. For the *EGFR* mutation detection, *EGFR* mutations of exon 18, 19, 20, and 21 were amplified using AmoyDx *EGFR* Mutations Detection Kit (Amoy Diagnostics, Shanghai, China) according to the principle of the amplification refractory mutation system. Positive or negative results were obtained if the criteria specified by the manufacturer’s protocol were met. All patients underwent a 5-year survival follow-up, and their survival data were recorded for subsequent analysis. This study was approved by the Ethics Committee of The Fourth People Hospital of Zibo (No. 00013269), and all participants provided written informed consent.

#### Dual-luciferase reporter assay

The binding sequences of miR-4429 to the 3’-UTR region of *EGFR* were predicted in starBase v2.0 platform (http://starbase.sysu.edu.cn/). The wild-type (WT)-*EGFR* sequence containing the miR-4429 binding site and mutant-type (MUT)-*EGFR* sequence was cloned into the pGL3 dual-luciferase reporter vector (Promega, Madison, WI, USA). Then, the WT*EGFR* and MUT*EGFR* vectors were co-transfected with miR-4429 mimic or mimic negative control (NC) into HEK293T cells using Lipofectamine 3000 reagent (Invitrogen, CA, USA) following the manufacturer’s instructions. The results were examined after 48 hours.

#### Total RNA extraction

Total RNA was extracted from the serum samples of NSCLC patients and healthy controls using TRIzol reagent (Invitrogen; Thermo Fisher Scientific, Inc.), and was then purified. The purity and concentration of the obtained RNA were evaluated by NanoDrop 2000 (Thermo Fisher Scientific, Inc.). The obtained RNA could be used for further analysis when the optical density (OD) ratio of 260 nm/280 nm was close to 2.0. The above operation was repeated 3 times. Then, the obtained RNA (1 μg) was reverse transcribed into cDNA using the PrimeScript RT Reagent Kit (TaKaRa, Japan), following the manufacturers’ instructions.

#### Real-time quantitative polymerase chain reaction (RT-qPCR)

Relative expression of miR-4429 and relative mRNA expression of *EGFR* was detected by RT-qPCR, which was performed using a SYBR green I Master Mix kit (Invitrogen, Carlsbad, CA, USA) on a 7500 Real-Time PCR System (Applied Biosystems, USA). The cel-miR-39-3p (spike in-control) was used as the internal reference of relative miR-4429 expression, and glyceraldehyde 3-phosphate dehydrogenase (GAPDH) was used as the internal reference of relative mRNA expression of *EGFR*. The primer sequences used for PCR were as follows: miR-4429 forward, 5’-GCCGAGAAAAGCTGGGCTGA-3’ and reverse, 5’-CTCAACTGGTGTCGTGGA-3’; cel-miR-39-3p forward, 5’-UCACCGGGUGUAAAUCAGCUUG-3’ and reverse, 5’-AACGCTTCACGAATTTGCGT-3’; *EGFR* forward, 5’-TTGCCGCAAAGTGTGTAACG-3’ and reverse, 5’-GTCACCCCTAAATGCCACCG-3’; GAPDH forward, 5’-TGGAAGGACTCATGACCACA-3’ and reverse, 5’-TTCAGCTCAGGGATGACCTT-3’. The PCR was performed 3 times. Relative miR-4429 expression and relative mRNA expression of *EGFR* were calculated by 2^ΔΔCT^ method [18].

### Ethical statement

The experimental procedures were all in accordance with the guideline of the Ethics Committee of The Fourth People Hospital of Zibo and has approved by the Ethics Committee of The Fourth People Hospital of Zibo. This study complies with the Declaration of Helsinki.

A signed written informed consent was obtained from each patient.

### Availability of data and materials

The data used to support the findings of this study are available from the corresponding author upon reasonable request.

### Statistical analysis

Data analysis results were shown as mean ± standard deviation (SD). All analyses were performed by SPSS 22.0 (IBM Corp.) and GraphPad Prism 7.0 software (GraphPad Software, Inc.). Comparisons between two groups of measurement data were performed using t-tests, and correlation analysis between variables was performed using the Pearson correlation analysis. The Chi-square test was used to analyze the association of miR-4429 with patients’ clinicopathological characteristics. Receiver operating characteristic (ROC) analysis was used to evaluate the clinical significance of serum miR-4429 in distinguishing NSCLC patients from healthy controls, and in distinguishing *EGFR* WT (*EGFR*-W) patients from *EGFR* MUT (*EGFR*M) patients. Kaplan-Meier curves were used to analyze the relationship of miR-4429 with the survival of NSCLC patients, and the log-rank test was used to analyze the difference between the two curves. Cox regression analysis was used to judge the prognostic value of miR-4429 in NSCLC patients. *p* < 0.05 indicated a statistically significant difference.

## RESULTS

### Circulating miR-4429 is negatively correlated with EGFR in NSCLC patients

**FIGURE 1:**
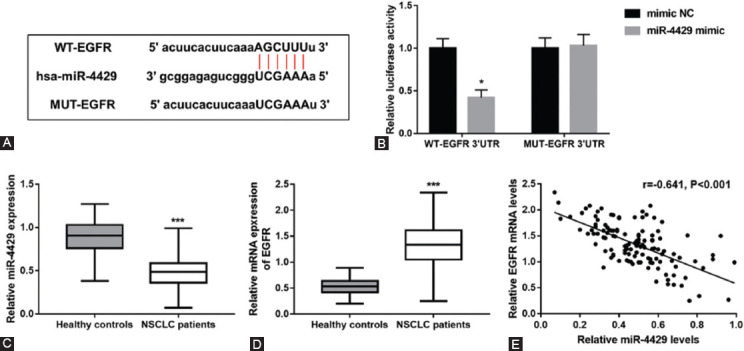
Correlation of miR-4429 with *EGFR* in NSCLC patients. (A) The binding sites of miR-4429 to *EGFR* was predicted by starBase v2.0 platform. (B) Relative luciferase activity in WT-*EGFR* group was decreased by miR-4429 mimic in HEK293T cells. (C) Serum miR-4429 expression in healthy controls and NSCLC patients. (D) Relative mRNA levels of *EGFR* in healthy controls and NSCLC patients. (E) Relative *EGFR* mRNA levels were negatively correlated with relative miR-4429 levels in the serum of NSCLC patients (r = -0.641, *p* < 0.001). *p < 0.05, ***p < 0.001 vs. mimic NC or healthy controls. *EGFR*, Epidermal growth factor receptor; WT, Wide-type; MUT, Mutant-type; NC, Negative control; NSCLC, Non-small cell lung cancer.

The results of bioinformatics prediction, shown in Figure 1A, exhibited the binding sites of miR-4429 to *EGFR*. Subsequently, the luciferase reporter assay results demonstrated that relative luciferase activity of the WT-*EGFR* group was decreased by miR-4429 mimic in HEK293T cells (*p* < 0.05), and no significant change was found in the luciferase activity of the MUT-*EGFR* group, indicating the direct binding between *EGFR* and miR-4429 (Figure 1B). Moreover, compared to healthy controls, NSCLC patients had significantly lower miR4429 levels (Figure 1C, *p* < 0.001) and significantly higher *EGFR* mRNA levels (Figure 1D, *p* < 0.001). Relative mRNA levels of *EGFR* were significantly negatively correlated with the relative miR-4429 levels in the serum of NSCLC patients (r = −0.641, *p* < 0.001; Figure 1E).

### Relationship between miR-4429 and NSCLC patients’ clinicopathological characteristics

As presented in Table 1, we found that miR-4429 was associated with tumor size (*p* = 0.033), *EGFR* mutation (*p* = 0.006), lymph node metastasis (*p* = 0.013) and TNM stage (*p* = 0.002) in NSCLC patients. Nevertheless, no association was found between miR-4429 and other characteristics, including age, gender, smoking, and histological subtypes (all *p* > 0.05).

**TABLE 1:**
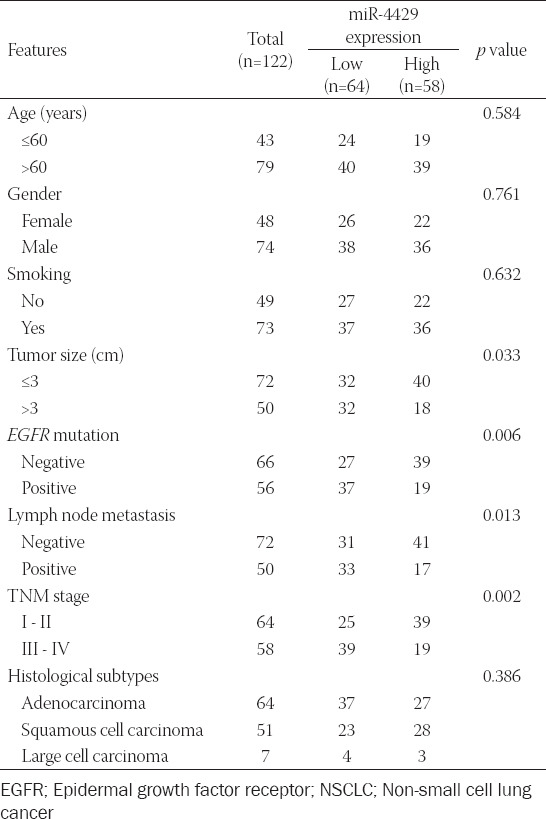
Relationship between miR-4429 and the clinicopathological characteristics of NSCLC patients

### Diagnostic performance of circulating miR-4429 in patients with NSCLC

**FIGURE 2:**
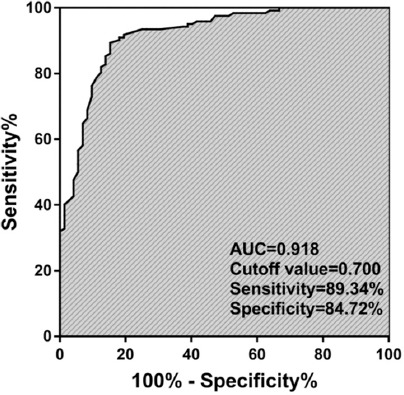
ROC analysis results indicated that serum miR-4429 had a high diagnostic value to screen NSCLC patients from healthy controls with an AUC of 0.918. AUC, Area under the ROC curve; ROC, Receiver operating characteristic; NSCLC, Non-small cell lung cancer.

ROC analysis results, shown in Figure 2, indicated that miR-4429 had high diagnostic potential to screen patients with NSCLC from healthy controls with an area under the ROC curve (AUC) of 0.918. At the optimal cut-off value of 0.700, the sensitivity and specificity were 89.34% and 84.72%, respectively.

### Prognostic value of miR-4429 in predicting the overall survival of NSCLC

**FIGURE 3:**
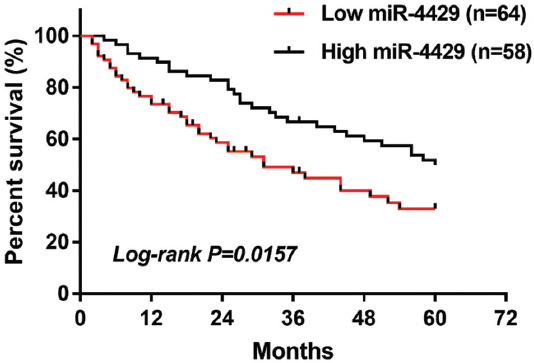
NSCLC patients with low levels of miR-4429 had lower overall survival than patients with high miR-4429 levels (log-rank *p* = 0.0157). NSCLC, Non-small cell lung cancer.

As presented in Figure 3, the survival curves indicated that NSCLC patients with low miR4429 levels had poor overall survival (log-rank *p* = 0.0157). The results of the Cox regression analysis of miR-4429 in predicting the overall survival of NSCLC were shown in Table 2. Univariate Cox regression analysis demonstrated that lymph node metastasis, TNM stage, and miR-4429 were associated with the overall survival of NSCLC patients. Then, the significant variables from univariate analysis results were included in multivariate Cox analysis. Multivariate Cox regression analysis results showed that TNM stage (hazard ratio [HR] = 2.372, 95% confidence interval [CI] = 1.606-3.008, *p* = 0.027) and miR-4429 (HR = 2.359, 95% CI=1.632-3.159, *p* = 0.009) were independently associated with the prognosis of NSCLC patients.

**TABLE 2:**
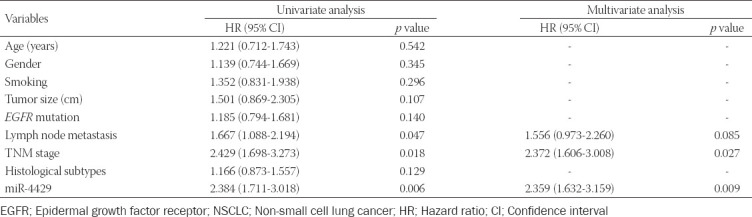
Cox regression analysis for miR-4429 to predict the overall survival of NSCLC

### Association of miR-4429 with EGFR mutation in patients with NSCLC

**FIGURE 4:**
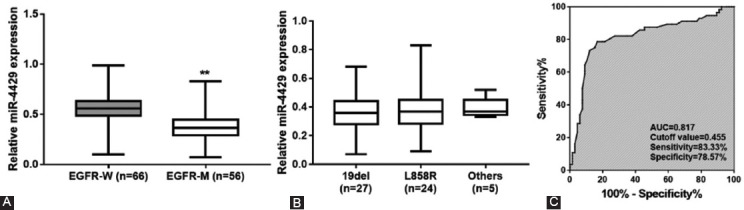
Relationship between miR-4429 and *EGFR* mutation in NSCLC patients. (A) Serum miR-4429 expression in patients with *EGFR*-M and patients with *EGFR*-W. (B) Serum miR4429 expression in patients with different types of *EGFR* mutations. (C) ROC analysis indicated the ability of serum miR-4429 to discriminate between *EGFR*-M patients and *EGFR*W patients. **p < 0.01 vs. *EGFR*-W; *EGFR*, Epidermal growth factor receptor; *EGFR*M, *EGFR* mutant-type; *EGFR*-W, *EGFR* wild-type; AUC, Area under the ROC curve; ROC, Receiver operating characteristic; NSCLC, Non-small cell lung cancer.

Fifty-six (45.9%) NSCLC patients with *EGFR* mutation were observed among the 122 NSCLC patients, including 27 (48.2%) cases of exon 19 deletions (19Del), 24 (42.9%) cases of exon 21 mutation (L858R), 3 (5.3%) cases of exon 21 mutation (L861Q) and 2 (3.6%) cases of S768I mutation in exon 21. Serum miR-4429 levels were significantly lower in *EGFR*-M patients than that in patients with *EGFR*-W (Figure 4A, *p* < 0.01). Additionally, no significantly different expression of miR-4429 was observed between patients with different types of *EGFR* mutations (Figure 4B, *p* > 0.05). Moreover, ROC analysis results indicated that serum miR4429 had a certain ability to differentiate between *EGFR*-M patients and *EGFR*-W patients (AUC = 0.817, Figure 4C).

## DISCUSSION

*EGFR* is an extensively studied and reported oncogene, thus, searching for molecules related to *EGFR* is necessary for NSCLC treatment. Currently, many miRs have been demonstrated to affect NSCLC by regulating *EGFR*. For example, Qi et al. have shown that *EGFR* is a direct target of miR-146a-5p, and it can reverse the effect of miR146a5p on NSCLC cell lines [19]. Besides, miR-145 has been reported to suppress the proliferation of human lung adenocarcinoma cells by regulating *EGFR* [20]. A study by Li et al. has revealed that miR-34a suppresses the progression of NSCLC tumors via targeting *EGFR* [21]. miR-218-5p suppresses NSCLC carcinogenesis by directly inhibiting *EGFR* [22]. In this study, we found the binding sites between miR-4429 and *EGFR* through starBase v2.0 platform and further demonstrated their direct binding by luciferase reporter assay. Moreover, downregulated miR-4429 and upregulated *EGFR* mRNA levels were found in NSCLC patients, and a negative correlation between miR-4429 and *EGFR* mRNA level was found in the serum of NSCLC patients. The above results indicated that miR-4429 is closely correlated with *EGFR* in NSCLC. The expression of circulating mRNA and miRNA in serum was measured in this study, including all mRNA and miRNA inside and outside cells. Thus, circulating mRNA and miRNA are not only contained extracellularly, not only from cancer cells, and their secretion includes active and passive secretion. Besides, mRNA digestion consists of total and partial digestion. Furthermore, miR4429 was found to be related to tumor size, *EGFR* mutation, lymph node metastasis, and TNM stage in NSCLC patients by the Chi-square test. miR-4429 has also been less frequent in other types of cancer, such as ovarian cancer [15], prostate cancer [23], and colorectal cancer [24]. Thus, we conclude that downregulated miR4429 may be involved in the progression of NSCLC.

Circulating miRs used as cancer biomarkers have been widely reported, including NSCLC. For instance, serum miR-203 has been reported to serve as a potential diagnostic and prognostic biomarker for acute myeloid leukemia [25]. A study by Sun et al. has shown that serum miR30a-5p may function as a new biomarker for the diagnosis and prognosis of colorectal cancer [26]. Serum miR-185 [27] and serum miR1246 [28] have been found to be used as diagnostic and prognostic biomarkers for NSCLC.

Given the significant decrease of miR-4429 in NSCLC, this study investigated its clinical significance in NSCLC. First, miR-4429 expression showed a high diagnostic value for screening NSCLC patients (AUC = 0.918), with a sensitivity of 89.34% and a specificity of 84.72%. Kaplan-Meier survival analysis results demonstrated that patients with high miR-4429 expression had significantly longer overall survival. Serum miR-4429 maintained significance as an independent prognostic indicator for the overall survival of NSCLC patients. In addition, miR-4429 has been found to serve as a potential diagnostic biomarker for biliary atresia [29] and is associated with the survival prognosis of other cancers, such as ovarian cancer [15] and clear cell renal cell carcinoma [30]. Thus, serum miR-4429 may function as a diagnostic and prognostic biomarker for NSCLC.

Oncogenic activating mutations of *EGFR* are associated with the development and progression of lung cancer. Over the past decades, *EGFR*-TKIs therapy, including gefitinib, erlotinib, and afatinib, has become the standard therapy for NSCLC patients with *EGFR* activating mutations [31]. Nevertheless, many NSCLC patients develop resistance to targeted drugs in approximately a year [32-34], resulting in poor prognosis and limited therapeutic efficacy. Thus, it is necessary to explore the molecules related to *EGFR* mutations of NSCLC. Some studies have found that miRs are associated with *EGFR* mutations of NSCLC [32,35,36]. The present study also found that miR-4429 is associated with *EGFR* mutation in NSCLC patients. Approximately 30% - 50% Asian and 10% - 15% Caucasian patients with NSCLC have *EGFR* mutation [37], which provides evidence for such a high *EGFR* mutation rate (45.9%) in this study. Therefore, miR-4429 may facilitate timely screening of patients with *EGFR* mutation to facilitate treatment with TKIs. In addition, we considered the reasons why miR-4429 was associated with *EGFR* mutation. It has been shown that there is a close association between *EGFR* expression level and *EGFR* mutation [38], thus, miR-4429 may be linked to the mutation of *EGFR* by regulating the expression of *EGFR*. However, how are they specifically related awaits further exploration.

Our study had some limitations. First, the sample size was relatively small, and our findings need further validation in large cohorts. Second, the molecular mechanism underlying the role of miR-4429 in NSCLC needs further exploration. Third, the lack of *in vitro* experiments and *in vivo* models to support this study’s conclusion is also a limitation of the study, which should be performed in future studies. Fourth, our study did not screen the entire *EGFR* gene (just exons 18-21). Fifth, learning if miR-4429 would also be changed in ethylenediaminetetraacetic acid (EDTA)-treated plasma samples can contribute to the potential use of miR-4429 as a biomarker, which will be conducted in the future study. NSCLC patients also harbor other genomic alterations, such as ALK1, ROS1, HER2, BRAF, KRAS, and NTRK mutations. Thus, we will explore the molecules associated with other genomic alterations of NSCLC patients to provide targets for NSCLC.

## CONCLUSION

Our study indicates that serum miR-4429 is decreased in NSCLC patients and may serve as a potential biomarker for the diagnosis and prognosis of NSCLC. In addition, there is a correlation between miR-4429 and *EGFR* mutation in NSCLC patients. Thus, our findings may provide a new effective target for the clinical therapy of NSCLC patients.
